# Preserved antiviral adaptive immunity following polyclonal antibody immunotherapy for severe murine influenza infection

**DOI:** 10.1038/srep29154

**Published:** 2016-07-06

**Authors:** Natalie E. Stevens, Antoinette Hatjopolous, Cara K. Fraser, Mohammed Alsharifi, Kerrilyn R. Diener, John D. Hayball

**Affiliations:** 1 Experimental Therapeutics Laboratory, Hanson Institute, and Sansom Institute, School of Pharmacy and Medical Science, University of South Australia, Adelaide, SA, Australia; 2 Preclinical, Imaging and Research Laboratories, South Australian Health and Medical Research Institute, Gilles Plains, Adelaide, SA, Australia; 3 Vaccine Research Group, Department of Molecular and Cellular Biology, School of Biological Sciences, The University of Adelaide, Adelaide, SA, Australia; 4 Robinson Research Institute, Discipline of Obstetrics and Gynaecology, School of Medicine, The University of Adelaide, Adelaide, SA, Australia

## Abstract

Passive immunotherapy may have particular benefits for the treatment of severe influenza infection in at-risk populations, however little is known of the impact of passive immunotherapy on the formation of memory responses to the virus. Ideally, passive immunotherapy should attenuate the severity of infection while still allowing the formation of adaptive responses to confer protection from future exposure. In this study, we sought to determine if administration of influenza-specific ovine polyclonal antibodies could inhibit adaptive immune responses in a murine model of lethal influenza infection. Ovine polyclonal antibodies generated against recombinant PR8 (H1N1) hemagglutinin exhibited potent prophylactic capacity and reduced lethality in an established influenza infection, particularly when administered intranasally. Surviving mice were also protected against reinfection and generated normal antibody and cytotoxic T lymphocyte responses to the virus. The longevity of ovine polyclonal antibodies was explored with a half-life of over two weeks following a single antibody administration. These findings support the development of an ovine passive polyclonal antibody therapy for treatment of severe influenza infection which does not affect the formation of subsequent acquired immunity to the virus.

Influenza infection claims approximately 250,000 lives worldwide annually and remains a significant burden on public health systems^1^. The majority of deaths from influenza infection occur in at-risk populations which include the immunocompromised, infants and the elderly, all which exhibit suboptimal responses to vaccination^2^,^3^ and are at higher risk of ‘severe’ influenza infection^4^. Severe influenza is defined as influenza infection accompanied by complications that require hospitalisation, and is attributed to over 3.4% of all critical hospitalisations during influenza season^1^. Current treatment for severe influenza generally incorporates supportive care and antiviral medications such as oseltamivir (Tamiflu^TM^) or zanamivir (Relenza^TM^)^5^, however viral resistance to such medications is increasing, and is most often acquired by infective viruses during hospitalisation and treatment^6^, which is a significant risk for immunocompromised patients hospitalised for extended durations^7^.

Vaccination is the most widely-used strategy to combat the morbidity and economic burden of influenza, with severe infection generally associated with failure to vaccinate^8^ or reduced efficacy of vaccination in immunocompromised populations^2^,^9^. Effective seasonal influenza vaccines elicit neutralising antibodies (Abs) against strain-specific glycoproteins, in particular haemagglutinin (HA) which is considered the major neutralising determinant of influenza^10^. However differences between the predicted HA incorporated into seasonal vaccines and the actual HA expressed by circulating strains can impact vaccine efficacy resulting in increased transmission and a higher burden of severe influenza infections^3^,^11^. It is clear therefore, that strategies beyond seasonal vaccination and antiviral medications should be explored to combat the morbidity and mortality of influenza infection, particularly during critical hospitalisation periods^12^.

Passive immunotherapy using neutralising Ab may be the ideal rapid treatment strategy for influenza infection that functions independently of the individual’s immunocompetency^12^ as highlighted by previous animal and human studies^13^,^14^,^15^. Furthermore, a recent multicentre double-blind randomised controlled trial has found that hyperimmune intravenous immunoglobulin (IVIG) prepared from donors exposed to pandemic H1N1 influenza significantly reduced viral load in infected individuals compared to IVIG prepared from donations received before the 2009 pandemic^16^. In a similar manner, further studies have shown reduced mortality in patients treated with convalescent plasma^17^. Animal studies have shown efficacy of anti-influenza enriched IVIG preparations in protecting immunodeficient mice from morbidity and mortality following influenza infection^18^, which indicates that this may be an effective strategy for use in immunocompromised patients. Despite clinical efficacy, the use of human-derived Ab therapies have significant logistical challenges including lengthy time periods for identification and screening of potential serum donors, and validation of sera and subsequent Ab products^19^. Consequently, current guidelines do not recommend IVIG or convalescent sera as a therapy for severe influenza^5^. Alternatively, passive immunotherapies utilising humanised monoclonal Abs (mAbs) have been developed for influenza prophylaxis and treatment, and whilst mAbs targeting HA have potent neutralising capacity, they are often not cross-reactive against multiple strains and may prompt the development of resistant mutants^20^. In contrast, mAb strategies that target highly conserved residues on the M2 ion channel protein are cross-reactive and demonstrate *in vivo* efficacy in experimental models^21^ however these are generally thought to function through the instigation of antibody-mediated cell cytotoxicity rather than direct viral neutralisation^20^,^22^. As such, the efficacy of these mAbs in immunocompromised individuals may be reduced.

Polyclonal Abs (pAbs) can overcome these deficiencies by their intrinsic ability to target multiple epitopes, which can increase cross-reactivity and reduce the development of resistant strains^23^,^24^. Approaches using pAbs sourced from animal sera are advantageous as large amounts of pAb can be collected and batched for reduced variability. Furthermore, pAbs have simplified screening procedures compared to human-derived Abs. Ovine pAbs are particularly efficient and cost-effective to produce, and exhibit reduced immunogenicity compared to those sourced from horses or other large mammals^25^. Though ovine-origin products carry a risk of contamination with transmissible prion proteins, sourcing of ovine serum from countries free from known prion disease such as Australia and New Zealand can remove this risk, and thus commercially available ovine pAb products are commonly sourced from regions such as these. Ovine pAb fragments have been used with success in the clinic, and are currently available as critical care therapies for life-threatening digoxin toxicity (DigiFab™, DIGIBIND™)^26^ and crotalid snake envenomation (CroFab™)^27^.

We have previously shown that ovine serum pAbs elicited against HA have potent neutralising capacity *in vitro* and are protective in an *in vivo* murine influenza model^13^. However, any Ab administered to prevent or treat influenza must not prevent the formation of adaptive immunity necessary for enduring protection against reinfection with future circulating strains, and this has not been explored in the context of foreign pAb delivery. Therefore the aims of this study were to explore the role of ovine anti-HA pAb therapy in a mouse model of severe influenza and to assess the effect on subsequent influenza-specific adaptive responses. It was demonstrated that ovine purified pAb treatment can effectively reduce mortality in the murine model and does not impede the development of protective humoral or cell-mediated responses to influenza.

## Methods

### Animals

Female 6–8 week old BALB/c mice were purchased from Laboratory Animal Services, The University of Adelaide and housed in IVCs under specific pathogen-free facility conditions with food and water provided *ad libitum*. All animal experiments were approved by the University of South Australia Animal Ethics Committee, and conducted in accordance with National and Institutional ethical and regulatory guidelines.

### Purification of ovine polyclonal anti-HA from serum

Sheep were immunised with baculovirus-produced recombinant HA (rHA) derived from PR8 influenza with serum stored at −20 °C as previously described^13^. Thawed serum was filtered through glass wool and pAb precipitated by addition of saturated ammonium sulfate solution to 45% (v/v). After centrifugation (20 min, 10,000 × g) pelleted protein precipitate was dissolved in milliQ water and dialysed into PBS before application to a Protein G agarose column (Thermo Fisher Scientific). Bound IgG was eluted with 0.1 M glycine (pH 2.5) and dialysed into PBS before concentration determination via BCA assay (Thermo Fisher Scientific) according to manufacturer’s instructions. Concentrated IgG was subsequently analysed for purity via SDS-PAGE. Control serum was sourced from non-immunised sheep and total IgG purified as described.

### Murine model of influenza infection

Groups of mice were administered purified pAbs (25 mg/kg) or PBS either via intraperitoneal (IP) injection (500 μL) or intranasal (IN) inhalation (32 μL) under anaesthetic. This dose was selected empirically from a pilot study (data not shown). For severe influenza infection, mice received IN challenge (32 μL) with PR8 H1N1 (500 TCID_50_) or A/PC H3N2 (3000 TCID_50_) influenza virus that had been purified from hen egg amniotic fluid as previously described^28^. Viral inocula were diluted on the day of use from freshly thawed viral stocks and untreated controls were run in parallel with all treated groups to ensure that the dose of virus administered caused an infection requiring euthanasia within 6–10 days. All challenged mice were provided with soaked food and sunflower seeds to support them through the associated pain and distress of infection, and were assessed daily for clinical symptoms of infection which included weight loss, ruffled coat, wheezing or abnormal respiration, hunched posture and reluctance to move. A clinical score of 5 or 20% weight loss was used to identify those mice reaching predetermined endpoints requiring euthanasia. Weight loss was the primary indicator for euthanasia in this study as most mice did not reach clinical thresholds.

### Haemagglutination-inhibition assay (HAI)

All HAI assays were performed as previously described^13^. Briefly, purified pAb (500 μg/mL; 30 μL) was pipetted into duplicate wells of a round-bottom 96-well plate and serially diluted two-fold in PBS before the addition of either PR8 influenza virus (5 haemagglutination (HA) units in 30 μL) to all wells. After 30 minutes incubation at room temperature, 0.5% (v/v) chicken red blood cells (cRBC) in PBS (30 μL) was added to each well and gently mixed. Plates were visualised over a light box after 45 minutes. The endpoint HAI titre was recorded as the highest dilution of test sample that was able to completely inhibit the agglutination of cRBC by virus in duplicate wells.

### ELISA analysis

Specific ELISA methods were employed to measure binding of anti-HA Abs against PR8 or A/PC virus and to quantify circulating anti-influenza Abs, anti-ovine Abs or ovine pAbs in mouse sera. Combinations of coating and detection Ab are summarised in Table 1. Briefly, EIA/RIA high-binding ELISA plates (Corning) were coated with 100 μL of the relevant coating protein diluted in 100 mM NaHCO_3_ buffer (pH9) and incubated overnight at 4 °C. Plates were blocked with 2% (w/v) BSA in PBS (1 hour, 37 °C) and the relevant samples diluted 1:2 for Ab quantification, or serially diluted for endpoint analysis in 1% (w/v) BSA in PBS with 0.05% Tween (PBS-T). After 3 washes with PBS-T (200 μL), diluted samples or serially diluted ovine pAb standards (32,000–2 ng/mL in PBS-T) were added to duplicate wells (100 μL) before incubation (2 hour, 37 °C). Plates were washed as before and bound serum Abs were detected by addition of the relevant detection Ab (100 μL) followed by incubation for 1 hour (37 °C). The plates were subsequently developed with OPD substrate (Sigma; 100 μL), the reaction stopped with 3 M HCl (50 μL), and the absorbance read at 490 nm. For endpoint ELISA methods, endpoint absorbance values were defined as the mean absorbance plus two standard deviations of negative control samples diluted 1:100.

### *In vivo* cytotoxic lymphocyte (CTL) assay

The ability of mice to mount a cell-mediated response to influenza infection was assessed using an *in vivo* cytotoxicity assay. Splenocytes from a donor mouse were harvested and RBC subsequently lysed. Washed splenocytes were pulsed with K^d^-restricted influenza nucleoprotein-derived peptide TYQRTRALV (NPP) before two washes in endotoxin-free PBS followed by CellTrace™ Far Red staining (0.1 μM) for 20 minutes at 37 °C. Cells were subjected to two more washes and mixed at a 1:1 ratio with unpulsed control cells stained with CFSE (3 μM; 10 minutes at 37 °C). Optimal staining was confirmed by flow cytometry (BD FACSCanto II) before transfer into infected or control mice via tail vein injection (8–10 million cells per mouse). Spleens from recipient mice were harvested 18 hours later and single cell preparations were assessed for percentage of FarRed and CFSE cells remaining via flow cytometry and FACSDiva software. Percent cell specific lysis was calculated using the following equation: % specific lysis = [1−(infected mouse %CFSE^pos^: %FarRed^pos^/control mouse %CFSE^pos^: %FarRed^pos^)] × 100.

### Statistical analyses

Statistical analyses were performed using GraphPad Prism V5 software. Survival curves were compared using the Mantel-Cox (log-rank) test. ELISA endpoints and *in vivo* CTL data were compared using two-tailed unpaired T-tests. Statistical significance was defined as P ≤ 0.05.

## Results

### Prophylactic administration of ovine anti-HA pAbs protects mice against lethal influenza infection

**Figure 1:**
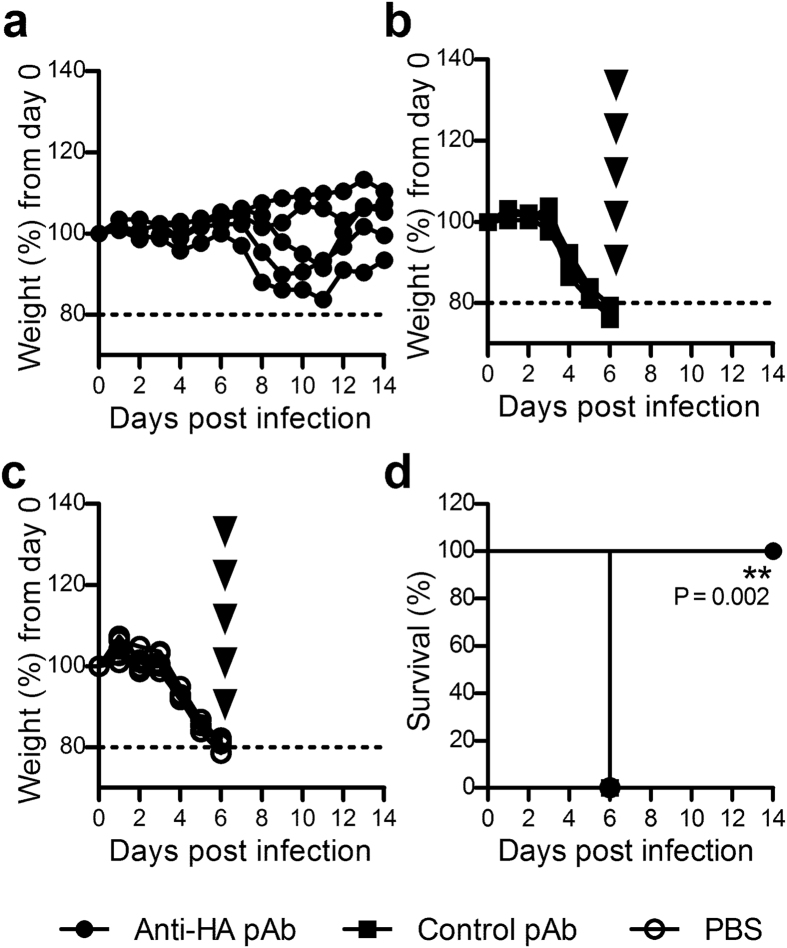
Administration of ovine anti-HA pAbs protects against subsequent lethal influenza infection. Mice were prophylactically administered (25 mg/kg in 500 μL) ovine anti-HA pAbs (**a**), control pAbs (**b**) or PBS (**c**) via the intraperitoneal route. Twenty-four hours later mice were challenged with 500 TCID_50_ (32 μL) PR8 influenza. The mice were closely monitored for weight loss (**a–c**) and euthanized according to predetermined humane endpoints (indicated by arrowheads). Data is presented as the weight loss of individual mice from infection day (n = 5). Corresponding survival curves (**d**) were analysed using the Mantel-Cox test to compare anti-HA treated to PBS treated and control treated mice. Significance is denoted as thus: ** = P < 0.01.

Previous studies have established the neutralisation capacity of generated whole ovine anti-HA antisera *in vitro* and *in vivo*^13^, however it remained necessary to confirm the potent neutralising capacity of the now purified IgG *in vivo.* To assess this, groups of mice were intraperitoneally administered purified ovine anti-HA pAbs, control pAbs, or PBS, and twenty-four hours later challenged intranasally with a lethal dose of PR8 influenza. Mice that had received anti-HA pAbs exhibited reduced weight loss (Fig. 1a) and mild signs of infection from days 2–6 (Supplementary Figure S1) and variable weight loss after day 7. However, all resumed weight gain after day 11, and none required euthanasia. In contrast, administration of control ovine pAbs or PBS could not control influenza infection which led to rapid weight loss from day 3 and required euthanasia by day 6 (Fig. 1b,c respectively). Thus a statistically significant survival advantage was conferred to the group of mice that received anti-HA ovine pAbs (Fig. 1d). These results clearly demonstrated the capacity of prophylactically-administered ovine anti-HA pAbs to completely protect against the fatal effects of a lethal influenza virus infection *in vivo*.

### Ovine anti-HA pAbs can reduce mortality in an established influenza infection

**Figure 2:**
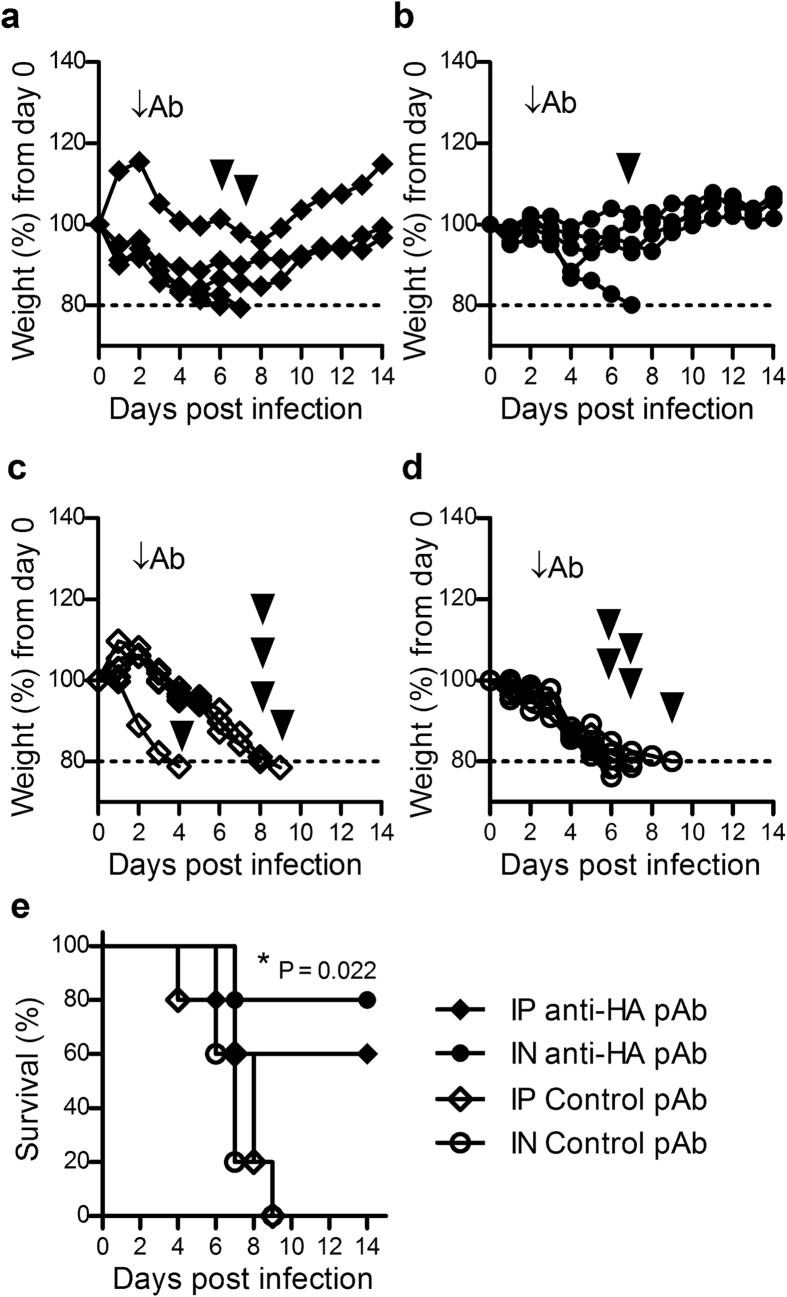
Ovine anti-HA pAb can treat established influenza infection. Mice were intranasally infected with 500 TCID_50_ (32 uL) PR8 influenza. Forty-eight hours later mice were administered anti-HA pAbs (25 mg/kg) via intraperitoneal injection (**a**), or intranasal administration (**b**). Control groups were intraperitoneally (**c**) or intranasally (**d**) administered control pAb. The mice were closely monitored for weight loss (**a–d**) and euthanized according to predetermined humane endpoints (indicated by arrowheads). Data is presented as weight loss of individual mice from infection day (n = 5). Survival curves (**e**) of treated groups were compared to control groups using the Mantel-Cox test. Significance is denoted as thus: * = P < 0.05.

The use of passive Ab immunotherapy to treat influenza infection would be of greatest benefit if administered as soon as possible after exposure, however in practice a diagnosis would not normally be made until after clinical symptoms were observed. Viral shedding in this murine model peaks two days after infection^29^,^30^ which correlates with an established infection in humans triggering symptom development^31^. Therefore, the therapeutic capacity of ovine anti-HA pAbs were assessed 48 hours after influenza challenge. Furthermore, as the route of administration can impact the effectiveness of Ab therapies^32^, two different routes were employed for this study. Whilst therapeutic administration of pAbs did not completely protect mice from reaching humane endpoints requiring euthanasia after infection, those mice that received ovine anti-HA pAbs via intraperitoneal injection (Fig. 2a) or intranasal administration (Fig. 2b) exhibited reduced weight loss compared to control mice that received control pAbs delivered systemically (Fig. 2c) or intranasally (Fig. 2d). These observations were also reflected in the corresponding clinical scores (Supplementary Figure S2). Of note, an unusual pattern of weight loss was observed in one mouse administered Ab via intraperitoneal injection (Fig. 2a), however no physiological anomaly was detected in this mouse while normal signs of infection were present and thus all the data pertaining to this mouse has been included. Analysis of overall survival (Fig. 2e) indicated significantly increased survival in intranasally-treated mice compared to the control groups (P = 0.022), although a survival benefit was also suggested in the intraperitoneal anti-HA pAb treated mice (P = 0.142). In a similar manner, mice that received antibodies by the intranasal route exhibited reduced severity in clinical symptoms of infection compared to antibodies delivered intraperitoneally (Supplementary Figure S2). These results support the development of a passive immunotherapeutic to treat established influenza infection, with treatment potentially more potent when administered intranasally.

### Anti-HA pAb treatment does not inhibit the formation of adaptive immune responses

**Figure 3:**
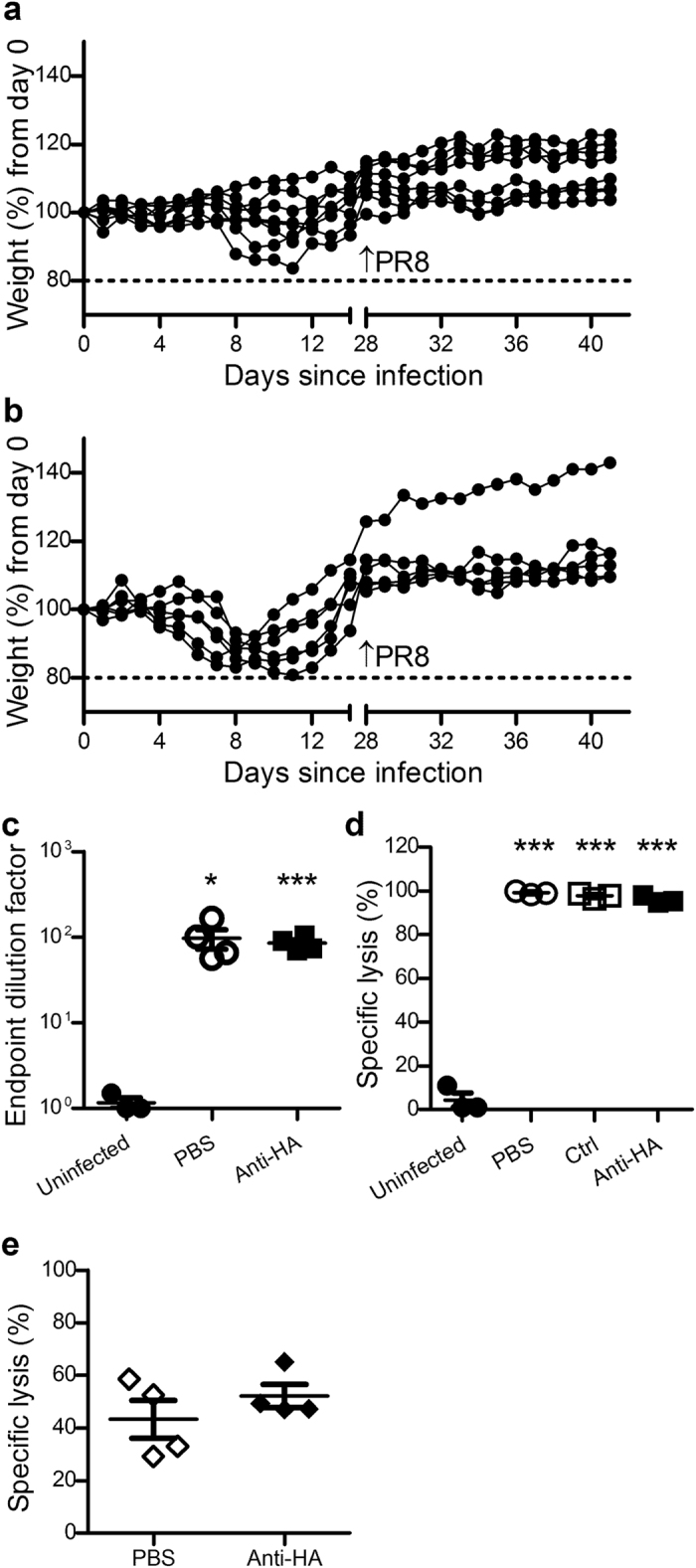
Prophylactic administration of anti-HA pAb does not inhibit the formation of protective adaptive immune responses. Mice that had survived influenza challenge via prophylactic intraperitoneal administration of anti-HA pAbs (25 mg/kg, n = 8; (**a**) or survived a sub-lethal challenge of 50 TCID_50_ PR8 influenza (n = 6; (**b**), were intranasally administered a lethal influenza dose of 500 TCID_50_ (32 μL) PR8 Influenza four weeks after their initial challenge. Data is presented as individual percent weight loss (**a–b**) depicting combined data from two separate experiments. For assessment of adaptive responses, serum samples from challenged mice prophylactically administered anti-HA pAbs or saline, or uninfected mice, were analysed by ELISA for PR8-specific IgG (**c**). For CTL responses, mice were prophylactically administered ovine anti-HA or control pAbs, or PBS and twenty four hours later infected with 250TCID_50_ PR8 influenza. Nine days later mice received 8–10 million CTL target cells IV and were assessed for specific killing capacity 18 hours later (**d**). Mice infected with A/PC influenza were also subjected to *in vivo* CTL as described (**e**). Data are representative of mean ± SEM (c–e). Ab titre and CTL data were compared using two-tailed Student’s t test; experimental groups were compared to uninfected controls. Significance is denoted as thus: * = P < 0.05, *** = P < 0.001.

A passive immunotherapeutic approach to treat severe influenza infection should not prevent the formation of endogenous antigen-specific immune responses against the infective strain. To investigate whether treatment with ovine anti-HA pAbs impacts on the development of long-term protective immunity, a rechallenge model was developed. Mice were prophylactically administered anti-HA pAbs twenty-four hours before administration of a lethal dose of PR8, while a second experimental group received a sub-lethal dose of PR8 with no pAb treatment. All mice recovered from infection and were rechallenged on day 28 with a lethal dose of PR8. Mice that had received initial protection by ovine anti-HA pAbs administration, survived a second lethal challenge without exhibiting the clinical signs associated with infection (Fig. 3a). The sub-lethal control group also survived a second lethal challenge without any significant morbidity (Fig. 3b). To ascertain the adaptive immune responses that may have contributed to this sustained protection, samples collected fourteen days post-infection from anti-HA pAb and PBS administered mice were assessed for PR8-reactive murine IgG via ELISA. Equivalent Ab responses were evident in anti-HA and PBS treated groups and were significantly higher than in uninfected animals (Fig. 3c). To measure cytotoxic T lymphocyte (CTL) responses, mice administered anti-HA pAbs, control pAbs or PBS were infected with a sub-lethal dose of PR8 influenza and an *in vivo* CTL performed ten days later. CTL responses to HA-specific targets from anti-HA pAb, control pAb and PBS treated groups were all of similar intensity, and all significantly higher than uninfected controls (Fig. 3d). However, since previous studies have shown that the formation of adaptive immunity to influenza is influenced by the number of viral particles^30^, and since anti-HA pAbs act to directly neutralise virions, responses between pAb-treated and untreated mice may have been confounded by this difference in viral load. To investigate this possibility, an alternate influenza strain with a disparate HA residue was employed. Mice were prophylactically administered anti-HA pAb or PBS and challenged with a sublethal dose of A/PC (H3N2) influenza with analysis of CTL responses performed ten days later. No significant difference in *in vivo* killing capacity between the groups was observed (Fig. 3e), indicating that the reduction in influenza symptoms, and thus viral load, did not impact on the generation of CTL responses in this model. Further confirmation was obtained by analysing serum samples from preliminary studies in which different doses of anti-HA pAb were administered prior to influenza infection for the development of murine anti-PR8 Abs (Supplementary Figure S3). There was no trend towards increased or decreased Ab responses in mice that received higher quantities of anti-HA Abs.

### Ovine anti-HA pAbs are immunogenic and are more efficiently cleared following a second administration

**Figure 4:**
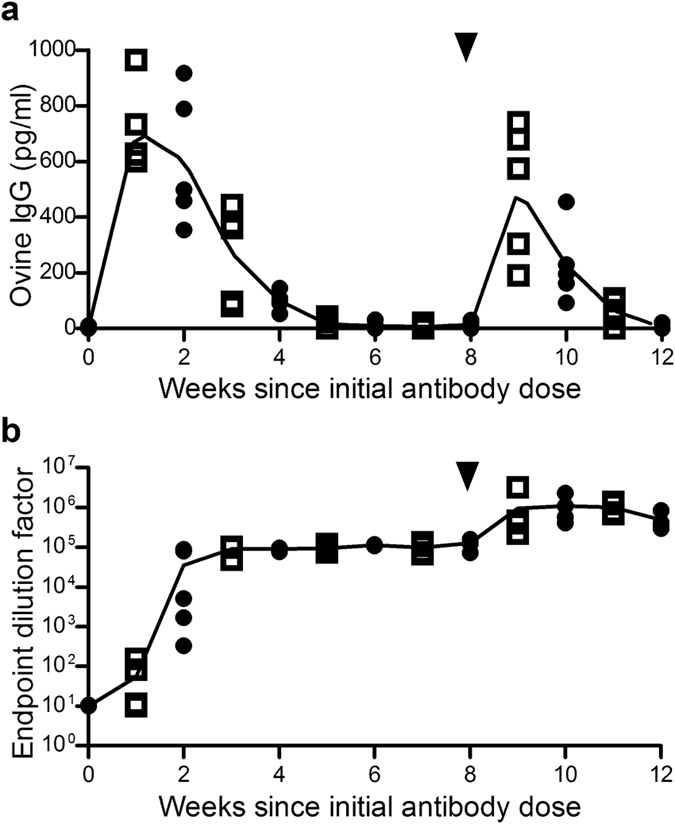
Kinetics of ovine pAb clearance and development of anti-treatment response. Two groups of BALB/C mice (n = 5) were administered ovine anti-HA pAbs (25 mg/kg) at day zero and eight weeks later (indicated by arrowheads), and bled every 14 days in two staggered timelines. Circulating ovine IgG was quantified via ELISA (individual values and mean depicted; (**a**) and mouse anti-ovine IgG response was quantified via endpoint ELISA (individual values and mean depicted; (**b**). Data is representative of two independent experiments.

In the event of an influenza epidemic, a passive immunotherapeutic approach targeting influenza would ideally provide protection for several weeks. In order to evaluate the longevity of ovine anti-HA passive immunotherapy, the half-life of circulating ovine pAbs was determined. Two groups of mice were dosed with anti-HA pAbs via intraperitoneal injection and administered a second dose eight weeks later. No observable morbidity was associated with the initial dose of the ovine pAb or re-dosing. Serum samples were collected every 14 days on a staggered timeline to achieve weekly measurements and analysed for the presence of ovine Abs, as well as the induction of murine anti-ovine IgG Abs as a measure of anti-treatment response. Seven days after the Ab administration, circulating ovine Abs (Fig. 4a) were detected at a maximum mean concentration of 700 pg/mL, which slowly declined over 5 weeks with a calculated half-life of 2.44 weeks. After a second pAb administration, ovine Abs were cleared more rapidly with the calculated half-life decreasing to 1.5 weeks. The development of murine anti-ovine IgG Abs was shown to occur 1–2 weeks after ovine pAb administration (Fig. 4b), with levels plateaued after three weeks. A second ovine pAb dose further increased murine anti-ovine IgG Ab levels, which suggested that the anti-treatment response contributed to the decreased half-life of circulating pAbs following a second administration.

### Ovine anti-HA pAbs cross-react with an antigenically distinct influenza strain *in vitro*, but are unable to effectively protect against infection *in vivo*

**Figure 5:**
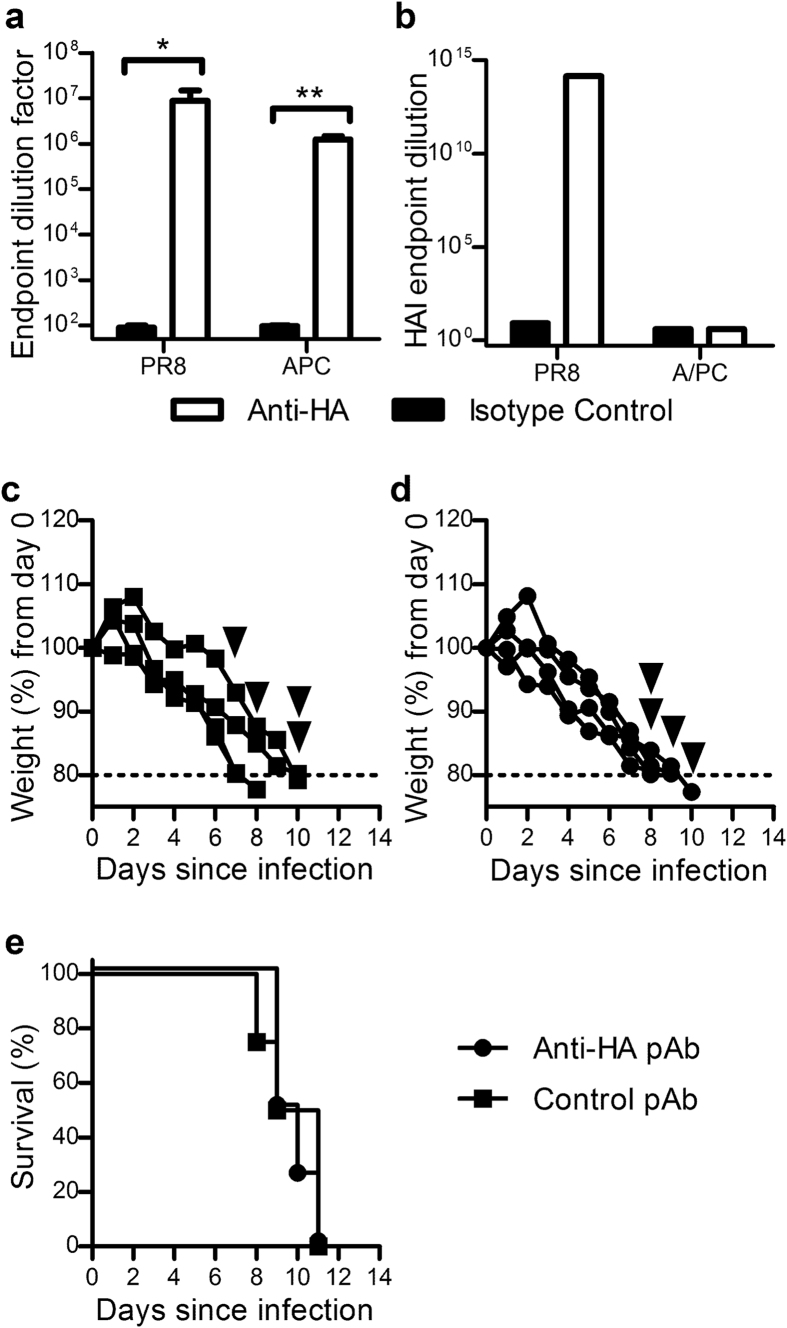
Cross-reactive ovine anti-HA pAbs do not exhibit neutralising capacity *in vitro* or protective capacity *in vivo* against a different influenza strain. Purified ovine anti-HA pAbs were analysed for reactivity against PR8 H1N1 and A/PC H3N2 influenza strains via ELISA and haemagglutination inhibition (HAI) assays. Absorbance values from ELISA results (mean ± SEM; (**a**) were analysed via Mann-Whitney analysis. Endpoint HAI titres are depicted in (**b**); data are representative of one assay performed in duplicate. To determine *in vivo* protective capacity against A/PC influenza, mice were administered either control pAb (**c**) or anti-HA ovine pAb (**d**) IP (25 mg/kg) and 24 hours later infected intranasally (32 μl) with A/PC (3000 TCID_50_). The mice were monitored closely and euthanized if 20% weight loss or a clinical score of 5 was achieved (indicated by arrowheads). Weight loss of individual mice (**c,d**) and group survival (**e**) are presented. Significance is denoted as thus: * = P < 0.05, ** = P < 0.01.

A polyclonal approach towards influenza-specific passive immunotherapy has the potential for cross-protective capacity of the administered pAbs against circulating variants of the infective strain, and in some cases, different strains of influenza^33^. Here, the ovine anti-HA pAbs were generated against full length PR8 (H1N1) HA, and cross-reactivity against an antigenically distinct H3N2 strain (A/PC) was assessed *in vitro* and *in vivo*. Purified anti-HA pAbs were shown to be capable of binding both viral strains via ELISA (Fig. 5a), suggesting that the production of ovine anti-HA pAb by immunisation of sheep with PR8 yielded some Abs capable of binding to A/PC. However, a haemagglutination-inhibition assay performed as a measure of direct neutralisation capacity against each strain clearly indicated that whilst the generated Abs were able to inhibit PR8-induced agglutination at a maximum of 10^13^ dilution, they were not able to inhibit agglutination by the A/PC viral strain at relatively low dilution of eight (Fig. 5b). In order to explore these conflicting results further, an *in vivo* infection model of A/PC influenza was employed where mice were administered anti-HA or control pAbs twenty-four hours before a lethal dose of A/PC. The mice administered control pAb exhibited sustained weight loss and succumbed to infection (Fig. 5c), as did those administered anti-HA pAb (Fig. 5d), thus indicating a lack of protection of anti-HA pAbs against A/PC influenza infection (Fig. 5e). These results taken together indicate that whilst the generated anti-HA pAbs are capable of binding to the A/PC strain *in vitro*, they are likely to have bound to sites on the A/PC HA that are not involved in host cell binding and entry, and therefore does not translate into protective capacity.

## Discussion

The number of people at higher risk of developing severe influenza complications is increasing due to the ageing population and expanding global burden of chronic disease. While vaccination and antiviral medications remain vital tools for the reduction of influenza mortality, vaccination in particular exhibits reduced efficacy in populations most vulnerable to severe infection leaving a significant number of deaths still attributed to influenza^4^,^34^. Therefore additional strategies to manage influenza infections are required. An influenza specific pAb with potent neutralising capacity may be particularly useful for severe influenza therapy as they exhibit rapid tissue penetration^23^ and could provide rapid specific protection to immunocompromised individuals who are at high risk of mortality. Additionally, polyclonal Abs boast straightforward and cost effective preparation methods^24^ and a good safety profile when administered to individuals with decreased immune capabilities^35^. Within this study, a potential role for passive immunotherapy via administration of influenza-neutralising ovine pAbs for the treatment of severe influenza infection was explored.

The efficacy of neutralising anti-HA pAb of human origin for protection against or treatment of influenza infection has been demonstrated in both animal^15^,^18^,^36^,^37^ and clinical^14^,^16^,^17^ studies. Previous studies, including our own, have demonstrated that hyperimmune anti-HA of ovine origin is efficacious in reducing mortality in a murine influenza model^13^,^15^ and this study confirmed that purified IgG from hyperimmune ovine serum retained protective and therapeutic capacity *in vivo* and *in vitro* (Figs 1 and 5). Administration of ovine pAb prior to infection reduced the severity of infection and reduced mortality compared to control mice. Importantly, administration of pAbs at 48 hours after challenge when peak viral shedding is observed was able to reduce mortality, with the intranasal route potentially more effective (Fig. 2). As viral shedding is higher in patients with comorbidities^38^, this was an important time point to assess. The ability to neutralise an established infection is crucial for a potential passive immunotherapeutic, as influenza-specific medication can only commence after the first symptoms of infection or positive blood test. In this therapeutic study, only those mice that received pAb intranasally exhibited a significant difference in clinical symptoms and overall survival from control groups. This is consistent with previous studies which showed that intranasal administration of anti-influenza Abs provided a survival advantage by significantly reducing clinical symptoms, weight loss and lung viral titres^15^,^37^,^39^. As this route of administration was also associated with higher Ab concentrations within the lung, it was postulated that a higher proportion of the delivered Abs were able to directly interact with virions in the nasal passages, as opposed to being distributed systemically via intraperitoneal injection before reaching the lungs^39^. Furthermore, previous studies, including our own with hyperimmune serum (Supplementary Figure S4), have indicated that intranasal Ab therapy requires less delivered Ab than systemic methods for the same outcome and can increase the longevity of protection^39^,^40^. These findings have important implications for the cost effectiveness of pAb products, which are often prohibitively expensive^41^, and may allow lower effective doses to be use and more affordable treatments. Intranasal pAb administration is also compatible with whole Ab and Ab fragments, which has been demonstrated experimentally for influenza^42^. Clinically, intranasal delivery of pAb products has been well tolerated and can provide adequate prophylaxis against respiratory infection^43^ though only intravenously administered human mAbs^21^,^44^ and IVIG^16^ have been effectively used for influenza treatment. Studies using an experimental human influenza model found that intravenous administration of anti-M2e Mab TCN-032 reduced symptom development and viral load when administered twenty-four hours following inoculation^21^, though whether intranasal administration may increase efficacy of treatments such as these remains to be investigated. Whilst intransally administered pooled immune globulin has been used unsuccessfully in the treatment of other respiratory infections^45^, no targeted Ab products have been trialled clinically for intranasal treatment of respiratory infections, including influenza, and remains to be investigated.

The current study also explored the effect of influenza-specific passive immunotherapy on the development of adaptive immune responses, which are important for protection against reinfection in at-risk populations. Previous studies have demonstrated that the presence of circulating influenza-specific Abs can inhibit humoral responses to a subsequent influenza vaccination in piglets^46^ and ferrets^36^. In the context of this study where ovine pAbs were assessed in murine influenza, the presence of foreign Abs might have favourable or non-favourable impacts on the generation of virus-specific responses. For example, Ab may effectively ‘mask’ available antigen from the immune system and prevent the generation of adaptive responses, as observed with the prevention of haemolytic disease of the newborn when pregnant mothers are prophylactically administered anti-D before sensitisation to the foetal D antigen can take place^47^. Alternatively, immune complexes formed *in situ* following administered foreign Ab may have an adjuvant-like function, as observed with historical use of antigen-antibody complexes to stimulate adaptive immunity^48^. In the current study, mice surviving infection via ovine anti-HA pAb administration exhibited sustained protection against influenza (Fig. 3) and symptoms of infection were largely absent upon reinfection. Influenza-specific antibody and cell mediated responses in mice administered pAb before infection were also assessed and both responses were found to be consistent with untreated groups. As symptoms of infection upon initial challenge were still evident even if administered anti-HA pAbs, it is postulated that in this setting passive immunotherapy served to neutralise some of the viable virions, effectively reducing but not eliminating viral load. Administration of passive pAb therapies clinically would occur only in an established infection, with a high viral load, thus viral-specific Abs would serve to attenuate rather than completely neutralise the infection. However, with a comparatively low viral load an alternate outcome may occur, and indeed studies have demonstrated that immunosuppressive effects are often present when Ab is administered in excess of antigen^49^. In the context of influenza, the use of ovine pAb in a sublethal model of PR8 infection (with a five-fold lower viral dose than the current study) was associated with reduced adaptive responses when infection was prevented via pAb administration one hour prior to viral challenge. However unlike our study where pAb were administered twenty-four hours prior to or forty-eight hours post challenge, in this scenario, virions were likely neutralised before attachment to host cells and antigen engagement by the immune system and thus the formation of an endogenous anti-PR8 immune response was prevented.

As part of the evaluation of an ovine pAb approach towards severe influenza treatment, within the current study the longevity and immunogenicity of passively administered pAbs in a murine model were assessed. The observed half-life of administered pAb was over two weeks and decreased after a second pAb administration. Correspondingly, anti-ovine responses which were evident from 1–2 weeks increased after re-administration and these likely contributed to the increased pAb clearance following re-administration. Though the administered ovine pAb were immunogenic, no hypersensitivity reactions were observed after pAb administration in this study. However, such reactions following administration of pAb of animal origin have been observed in the clinic^25^. Formulation of therapies as Fab or F(ab)_2_ fragments, thus removing the F_c_ portion, serve to reduce this risk but do not eliminate it. As such, administration of ovine pAb fragment therapies is closely monitored and appropriate precautions, including readiness with intravenous epinephrine and antihistamines, are taken in patients with a history of sensitivity to sheep proteins or previous administration of ovine pAbs^50^. Administration of ovine pAb also did not impact on the development of influenza-specific immunity in this model. Other studies have indicated that passive immunotherapies can perform an immunogenic ‘adjuvant-like’ function and increase adaptive immune responses to their targets. For instance, the clinical efficacy of intravenous immunoglobulin (IVIG) against a wide variety of conditions has been linked to immunomodulatory properties potentially via engagement of Fc-gamma immunoglobulin receptors^51^. An immunomodulatory adjuvant effect has also been observed in candidate anti-D mAbs developed for prevention of hemolytic disease of the newborn which enhanced rather than prevented endogenous anti-D immune responses^52^. It was proposed that Ab glycosylation may be the key to these adjuvant functions and this is supported by studies examining carbohydrate-dependent effector function of Abs^53^. In the context of influenza, these effector-functions could be harnessed to develop passive Ab therapies that not only neutralise influenza virions but enhance host anti-viral immunity, potentially via the formation of immunogenic immune complexes and engagement with Fc-gamma receptors. While no enhancement of adaptive responses was associated with ovine pAb administration in this study, whether whole ovine pAb are capable of instigating immunomodulatory effects through interaction with human Fc receptors remains to be explored.

While influenza-specific mAb therapies have been shown to be efficacious in animal^32^,^39^,^54^ and human^21^ studies, these monoclonal therapies may not be neutralising against a diverse range of viral strains^22^ and may prompt escape mutants to be generated^10^,^55^. A major advantage of a polyclonal approach to influenza treatment is the ability to target multiple epitopes to discourage escape mutants and engineer therapies that neutralise multiple strains of influenza. Within this study, pAbs were generated against a single recombinant HA from PR8, H1N1, and shown to have *in vitro* binding capacity against a H3N2 strain (A/PC) via ELISA analysis. However the pAbs did not exhibit functional neutralisation capacity against this strain (Fig. 5b) and thus were not cross protective. It may be that the H3N2-reactive pAbs evident by ELISA are targeted towards epitopes distant from the sialic-acid binding site responsible for host cell binding *in vivo* and haemagglutination *in vitro.* As pAbs in this study were elicited against full length monomeric HA, and fragmented or denatured virions may have been present in the ELISA method, it is possible that cross-reactive Abs were binding to stem region epitopes that were exposed in ELISA but are not exposed in trimerised HA on whole virus^56^. These non-neutralising Ab therefore did not impact on the course of infection in an *in vivo* H3N2 model (Fig. 5c–e). While cross-protective capacity between two strains was not evident within this study, strategies to increase cross-protective capacity could be employed for the generation of anti-influenza polyclonals. Such strategies could include immunisation of sheep with multiple influenza strains, repeated boosting and the application of superior adjuvants not approved for human use or simply combination of antisera elicited against different strains^23^,^57^,^58^.

The current study provides proof-of-principle for development of a passive immunotherapeutic based on ovine pAb for the treatment of established severe influenza infection. It also provides evidence that pAb therapies will not reduce adaptive anti-influenza responses which are crucial to ongoing protection from influenza infection. Further studies are however required to assess the *in vivo* viral neutralisation capacity of the therapy, particularly in the context of pAb fragmentation for clinical use. Through further development, strategies to decrease Ab immunogenicity, increase cross-protective capacity or increased immunomodulatory functions of the Abs could be employed to produce a purpose-built passive pAb therapy capable of controlling life-threatening influenza infections in immunologically-deficient populations.

## Additional Information

**How to cite this article**: Stevens, N. E. *et al*. Preserved antiviral adaptive immunity following polyclonal antibody immunotherapy for severe murine influenza infection. *Sci. Rep.* **6**, 29154; doi: 10.1038/srep29154 (2016).

## Supplementary Material

Supplementary Information

## Figures and Tables

**Table 1:** Antibodies used for ELISA assays.

Detected Analyte	Sample	Coating protein	Coating Concentration	Detection Ab	Concentration	Depicted in
Murine anti-PR8 IgG	Murine sera	PR8 virus	500 TCID_50_/mL in 100 mM NaHCO_3_	HRP-linked rabbit anti-murine IgG (Sigma; Cat #A9044)	1:10,000 in 1% BSA PBS-T	Figure 3c
Ovine IgG in circulation	Murine sera	Purified donkey anti-sheep IgG (Sigma; Cat # SAB3700721)	5 μg/mL in 100 mM NaHCO_3_	HRP-linked anti-sheep IgG (Sigma; Cat # A3415)	1:15,000 in 1% BAS PBS-T	Figure 4a
Murine anti-ovine IgG	Murine sera	Ovine pAb	10 μg/mL in 100 mM NaHCO_3_	HRP-linked rabbit anti-murine IgG (Sigma; Cat # A9044)	1:10,000 in 1% BSA PBS-T	Figure 4b
Ovine anti-PR8 IgG	Ovine pAb	PR8 virus	500 TCID_50_/mL in 100 mM NaHCO_3_	HRP-linked anti-sheep IgG (Sigma; Cat # A3415)	1:15,000 in 1% BAS PBS-T	Figure 5a
Ovine anti-A/PC IgG	Ovine pAb	A/PC virus	500 TCID_50_/mL in 100 mM NaHCO_3_	HRP-linked anti-sheep IgG (Sigma; Cat # A3415)	1:15,000 in 1% BAS PBS-T	Figure 5a

ELISA protocols were developed to assess the levels of murine anti-PR8 IgG, circulating ovine IgG or murine anti-ovine IgG in sera from infected or treated mice, or to assess the ability of ovine IgG to bind to PR8 and A/PC viruses.
