# Supplementary material for: Preserved antiviral adaptive immunity following polyclonal antibody immunotherapy for severe murine influenza infection
Source: Sci Rep. 2016 Jul 6;6:29154. doi: 10.1038/srep29154 (PMC4933909; doi:10.1038/srep29154)
Supplement: Supplementary Information [file srep29154-s1.pdf]

SUPPLEMENTARY INFORMATION

Preserved antiviral adaptive immunity following polyclonal antibody immunotherapy for severe murine influenza infection

Natalie E. Stevens[1], Antoinette Hatjopolous[1], Cara K. Fraser[2], Mohammed Alsharifi[3], Kerrilyn R. Diener[1,4], and John D. Hayball.[1,4]

[1] Experimental Therapeutics Laboratory, Hanson Institute, and Sansom Institute, School of Pharmacy and Medical Science, University of South Australia, Adelaide, SA, Australia;

[2] Preclinical, Imaging and Research Laboratories, South Australian Health and Medical Research Institute, Gilles Plains, Adelaide, SA, Australia;

[3] Vaccine Research Group, Department of Molecular and Cellular Biology, School of Biological Sciences, The University of Adelaide, Adelaide, SA, Australia;

[4] Robinson Research Institute, Discipline of Obstetrics and Gynaecology, School of Medicine, University of Adelaide, Adelaide, SA, Australia.

SUPPLEMENTARY FIGURE S1

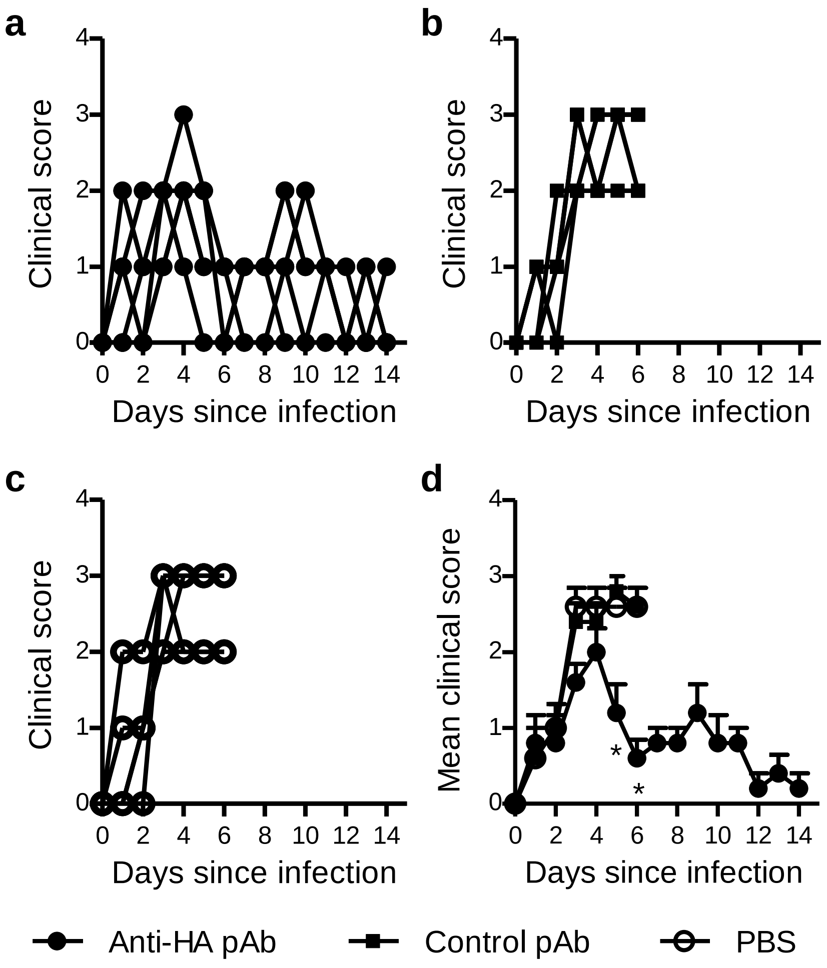

Supplementary Figure S1: Prophylactic anti-HA pAb intraperitoneal administration reduces symptoms of influenza infection. Mice (*n* = 5) were administered (a) ovine anti-HA or (b) control pAbs (25 mg/kg), or (c) PBS, via intraperitoneal injection twenty four hours prior to infection with 500 $TCID_{50}$ PR8 influenza. Mice were monitored for disease progression and attributed a clinical score daily. Scores of individual mice (a-c) and mean + SEM group scores (d) are depicted. Mean scores of anti-HA pAb administration group on days 5 and 6 were compared to control groups via Mann-Whitney analysis, where * = p>0.05.

SUPPLEMENTARY FIGURE S2

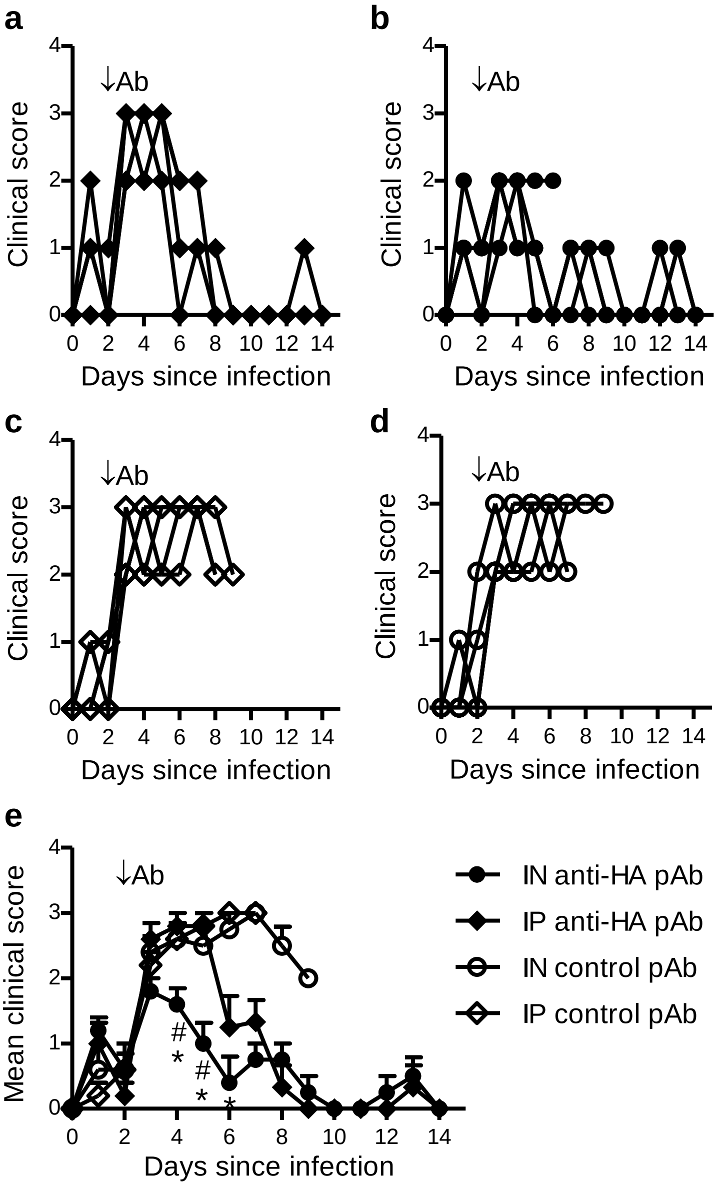

Supplementary Figure S2: Intranasal administration of anti-HA pAbs significantly reduce the clinical symptoms of influenza infection. Mice (n = 5) were infected with 500 $TCID_{50}$ PR8 influenza and forty-eight hours later were administered ovine anti-HA pAbs (a, b) or control pAbs (c, d) (25 mg/kg) via intraperitoneal injection (a, c) or intranasal instillation (b, d). Individual mice were monitored for disease progression and attributed a clinical score daily (a-d), with group mean + SEM of scores indicated in (e). Average scores of the intranasal administration group on days 4, 5 and 6 were compared to control Ab administration and intraperitoneal anti-HA administration via Mann-Whitney analysis, where * = p>0.05 compared to control groups, # = p>0.05 compared to IP anti-HA group.

SUPPLEMENTARY FIGURE S3

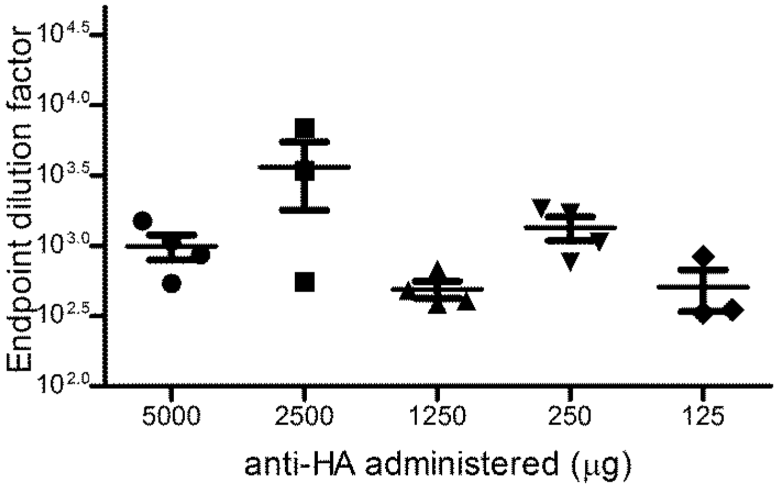

Supplementary Figure S3: Passively administered anti-HA pAbs do not inhibit the generation of murine anti-PR8 Abs upon influenza infection. Mice were intraperitoneally administered ovine serum containing anti-HA antibodies to the indicated dose, and twenty-four hours later were infected intranasally (32 μL) with 500 $TCID_{50}$ PR8 Influenza. Mice were bled 14 days following infection and anti-PR8 IgG titres were evaluated via endpoint ELISA. Data are presented as mean ± SEM.

SUPPLEMENTARY FIGURE S4

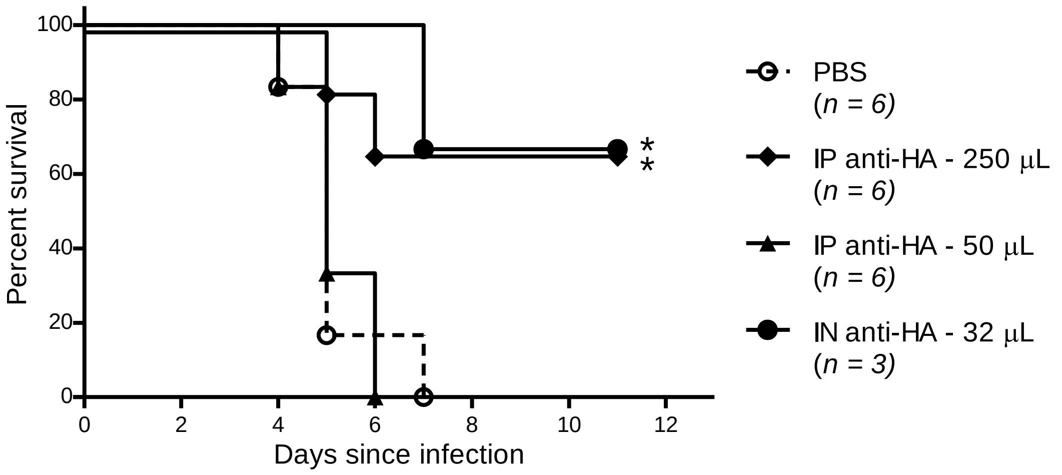

Supplementary Figure S4: Effective influenza treatment can be achieved from a lower dose of anti-HA hyperimmune serum administered through an intranasal route compared to an intraperitoneal route in a murine influenza model Mice (*n* = 3-6) were infected with 500 $TCID_{50}$ PR8 influenza and twenty four hours later were intranasally (IN) or intraperitoneally (IP) administered anti-HA hyperimmune serum or PBS. Mice were monitored and those reaching a predetermined endpoint of 20% weight loss were euthanized. Survival curves of treatment groups are depicted. Mantel-Cox survival analysis was performed to compare treatment groups to PBS administration group; significance between survival curves is denoted as thus: * = P<0.05.
